# A new genetic architecture for PHS resistance in rice: deciphering the epistatic interactions of three major QTL

**DOI:** 10.3389/fpls.2026.1778741

**Published:** 2026-02-11

**Authors:** Chang-Ju Lee, Tae-Heon Kim, Dong-Hyun Baek, Jingli Gao, Woo-Geun Park, Suk-Man Kim

**Affiliations:** 1 Department of Ecological and Environmental System, Kyungpook National University, Sangju, Republic of Korea; 2 Institute of Agricultural Science and Technology, Kyungpook National University, Daegu, Republic of Korea

**Keywords:** antagonistic epistasis, genetic architecture, GWAS, pre-harvest sprouting, rice

## Abstract

Pre-harvest sprouting (PHS), the premature germination of grains before harvest, threatens rice yield and quality under erratic climatic conditions. This study aims to investigate the genetic basis of PHS resistance by conducting a genome-wide association study (GWAS) on 182 diverse rice genetic resources representing multiple ecotypes using 289,569 high-quality single-nucleotide polymorphisms. Three major QTLs—*qRPH7, qRPH8, and qRPH11*—were identified using the complementary multi-locus models, Bayesian information and Linkage disequilibrium, iteratively Nested Keyway and Multi-Locus Mixed Model. *qRPH7* showed the strongest association, explaining up to 80% of phenotypic variance, and co-localized with *SDR4* and *qPH7*. Allelic combination analyses revealed that the *qRPH7–SDR4* and *qRPH7–qPH7* combinations conferred strong resistance, whereas *qRPH7* alone was insufficient. In contrast, *qRPH11* contributed additively to enhance resistance, while *qRPH8* displayed antagonistic epistasis that reduced resistance stability. Overall, PHS resistance is governed by a polygenic architecture involving both additive and epistatic interactions. These findings establish a new genetic architecture underlying PHS resistance in rice and propose a targeted breeding strategy through pyramiding *qRPH7* with *SDR4, qPH7*, and *qRPH11*. This study advances mechanistic insight into seed dormancy and sprouting while providing actionable resources to support marker-assisted selection and accelerate the development of PHS-resistant cultivars suited to climate change.

## 1 Introduction

Climate change has intensified unpredictable abiotic stresses, including heat waves and erratic rainfall, resulting in major crop yield losses, necessitating urgent intervention from agricultural research institutions (Lobell et al., 2011; Benitez-Alfonso et al., 2023). In rice, these climatic shifts promote pre-harvest sprouting (PHS) under hot, humid conditions during grain filling, leading to substantial production losses (Baek and Chung, 2014; Sohn et al., 2021). PHS, characterized by premature seed germination, reduces yield, lowers milling recovery, and degrades grain quality, ultimately posing a critical threat to farmer income and global food security (Zhu et al., 2019; Lee et al., 2020). In temperate *japonica* rice-growing regions, including Japan, Korea, and California (USA), PHS is projected to incur significant cumulative losses of USD 8–10 billion under extreme conditions and USD 4–5 billion under milder scenarios over the next decade (Lee et al., 2021). Mitigating these risks requires the use of diverse rice genetic resources to advance resistance breeding. Specifically, identifying resistant genetic resources and uncovering quantitative trait loci (QTLs) and candidate genes offer promising breeding strategies to minimize the adverse effects of climate change on rice production and ensure a stable food supply (Li et al., 2011; Lobell et al., 2011; Mizuno et al., 2018). However, most studies rely on biparental populations or focus narrowly on a few major-effect loci, such as *SDR4* and *qPH7* (Sugimoto et al., 2010; Lee et al., 2023). Although these approaches have yielded valuable insights, they may not fully capture the full extent of natural genetic variation present in diverse rice genetic resources, potentially limiting the development of durable resistance to PHS.

PHS resistance is closely linked to seed dormancy, a mechanism that prevents premature germination under unfavorable conditions (Bewley, 1997). Seed germination is regulated by both environmental factors, including temperature, moisture, and oxygen availability, and intrinsic hormonal signals (Née et al., 2017; Penfield, 2017; Klupczyńska and Pawłowski, 2021). Abscisic acid (ABA) induces dormancy, whereas gibberellins (GA) stimulate germination, with the ABA–GA balance largely determining seed fate (Finch-Savage and Leubner-Metzger, 2006; Finkelstein et al., 2008). ABA signaling operates through the PYR/PYL–PP2C–SnRK2 module to maintain dormancy, while GA induces germination by promoting DELLA protein degradation (Tyler et al., 2004; Umezawa et al., 2010; Ali et al., 2022; Lan et al., 2024). Dormancy release occurs through processes such as after-ripening or dry storage, which reduces ABA sensitivity, enhances GA responsiveness, and is accompanied by reactive oxygen species (ROS) accumulation and chromatin modifications (Liu et al., 2014). Structural and biochemical properties of the seed coat, including inhibitory compounds and physical barriers to water or oxygen, further contribute to dormancy maintenance and PHS resistance (Debeaujon et al., 2000). The husk, pericarp, and testa restrict water uptake, oxygen diffusion, and embryo expansion, closely linking these barriers to PHS resistance in rice (Roberts, 1961). Additionally, the seed coat contains germination-inhibitory compounds, such as phenolics and alkaloids, which reinforce dormancy through both physical and chemical inhibition (Chenyin et al., 2023).

Numerous genes and QTLs linked to seed dormancy and PHS resistance have been identified in rice. Among them, *Seed dormancy 4* (*SDR4*) is a key regulator that integrates ABA and GA signaling to reinforce dormancy and shows strong associations with PHS resistance across diverse genetic resources (Sugimoto et al., 2010). More recently, a major QTL for PHS resistance, *qPH7*, was identified using a recombinant inbred line population derived from Korean weedy rice, and fine-mapping localized it to a 210-kb interval (23.575–23.785 Mb) on chromosome 7 (Lee et al., 2023).

Beyond these loci, additional genetic determinants have been identified. For instance, *Rc (qSD7-1)*, which controls seed coat pigmentation, is consistently linked to dormancy and PHS resistance (Gu et al., 2003, 2004). *qSD12*, mapped in multiple biparental populations, contributes to natural variation in dormancy by promoting ABA accumulation in early developing seeds to induce primary dormancy (Gu et al., 2008, 2010). Carbohydrate metabolism-related loci such as *PHS8/ISA1* further highlight the role of endosperm composition in PHS regulation (Du et al., 2018). Regulatory genes involved in hormonal signaling also contribute to dormancy control. *OsVP1* functions as a central transcription factor coordinating ABA-mediated seed maturation and dormancy, while *qSD1*-*2*/*OsGA20ox2* encodes a GA biosynthesis enzyme that modulates GA levels and the dormancy-germination balance (Ye et al., 2015; Chen et al., 2021). The *OsDOG1L* gene family maintains dormancy through mechanisms similar to those of the Arabidopsis *DOG1* pathway (Bentsink et al., 2006; Wang et al., 2020). The major QTL *qLTG3–1* enhances low-temperature germinability by weakening embryonic tissues—improving germination under suboptimal temperatures (Fujino et al., 2008). Collectively, these studies show the polygenic complexity of PHS resistance in rice, integrating hormonal regulation, metabolic pathways, and structural seed traits that govern dormancy and germination. However, despite considerable progress in elucidating the genetic control of PHS resistance, studies largely focus on biparental populations or a few major-effect loci, limiting relevance to the broader genetic diversity of rice genetic resource. Furthermore, the polygenic and environmentally sensitive nature of PHS resistance, driven by the interplay of seed dormancy, hormone regulation, and structural traits, suggests that key components of its genetic architecture remain unresolved.

Therefore, this study aims to investigate PHS resistance by conducting a genome-wide association study (GWAS) on 182 rice genetic resources representing multiple ecotypes to capture natural allelic variation beyond the resolution of conventional linkage mapping. This study identifies novel QTL through GWAS and systematically examines their genetic interactions with previously reported loci such as *SDR4* and *qPH7*, thereby clarifying the complex architecture underlying PHS resistance. By highlighting allelic combinations with practical breeding value, the findings could provide mechanistic insights and actionable resources for marker-assisted selection, supporting the development of rice cultivars with stable PHS resistance under diverse climatic conditions.

## 2 Materials and methods

### 2.1 Plant materials

A panel of 182 rice genetic resources was used for phenotypic and genotypic evaluation to identify genomic regions associated with PHS resistance. The set of genetic resources comprised 106 *Japonica*, 35 *Indica*, 33 *Admixed*, 6 *Aus*, and 2 *Aromatic* types. Of the 182 rice genetic resources, 116 were obtained from the National Institute of Crop Science, and the remaining were sourced from the National Agrobiodiversity Center.

### 2.2 Field management

The experiment was conducted in 2024 at the Experimental Farm, College of Agriculture and Life Sciences, Kyungpook National University. Seedlings were transplanted at a spacing of 30 × 15 cm, with one seedling per hill. Fertilizer was applied at rates of 9.0–4.5–5.7 kg/10a (N–P_2_O_5_–K_2_O), following national crop fertilizer guidelines (National Institute of Agricultural Sciences, 2022).

### 2.3 Pre-harvest sprouting evaluation

PHS resistance was evaluated by recording the heading date of each rice genetic resource and harvesting the main panicle 40 days after heading, corresponding to an accumulated growing degree day value of 1,000 °C (Kang et al., 2018). To ensure phenotypic reliability, seed viability was assessed after the PHS evaluation by conducting an independent post-harvest germination test using harvested seeds. For each rice genetic resource, 30 seeds were placed in a Petri dish with three biological replicates and incubated at 25 °C for 7 days under standard germination conditions. All rice genetic resources used in this study showed germination rates above 70% in this test. Three biological replicates were included per genetic resource. Panicles were fully wrapped in tissue paper to facilitate moisture absorption and placed in stainless steel trays (325 × 265 × 63 mm). Samples were incubated in a growth chamber at 25 °C and 100% relative humidity for 7 days (Rural Development Administration, 2012). After incubation, the germination rate was calculated as the percentage of germinated seeds among the total number of filled seeds per panicle. The mean value of three replicates was used to determine the final PHS rate.

Seeds were considered germinated when the coleoptile visibly emerged from the hull, while unfilled or defective grains were excluded. Based on germination rates, PHS resistance was classified into five categories: degree 1 (≤ 20%), degree 3 (21–40%), degree 5 (40–60%), degree 7 (60–80%), and degree 9 (81% ≤) (Rural Development Administration, 2012). **Table 1** presents the classification criteria. Rice genetic resources with degrees 1 or 3 were considered resistant, while those with degrees 5, 7, or 9 were considered susceptible.

**Table 1:** Criteria for the classification of evaluating PHS severity.

Degree	Observation	Tolerance
1	≤ 20%	Highly Tolerant
3	21–40%	Tolerant
5	41–60%	Moderately Tolerant
7	61–80%	Susceptible
9	81% ≤	Highly Susceptible

### 2.4 Genotyping data collection and processing

Single-nucleotide polymorphism (SNP) genotyping was performed using the 580K Axiom Rice Genotyping Chip (580K_KNU chip), developed from eight genomic data sources (Kim et al., 2022). Genomic DNA samples were hybridized to the array and scanned on the GeneTitan^®^ platform, Affymetrix, Santa Clara, CA, USA. SNP calling was conducted with Genotyping Console v4.2, Affymetrix, Santa Clara, CA, USA, and further refined using the SNPolisher R package v3.0. SNPs were aligned to the IRGSP-1.0 (*japonica*), MH63RS2 (*indica*), and *Oryza rufipogon* reference genomes. High-quality SNP markers were selected for GWAS. They were filtered using the following criteria: minor allele frequency (MAF) > 0.05, missing rate < 0.02, heterozygosity rate < 0.05, removal of non-polymorphic SNPs, and sequencing depth > 10×. After filtering, 289,569 SNPs were retained for GWAS.

### 2.5 Genome-wide association study

GWAS was performed using two multi-locus models—Bayesian information and Linkage-disequilibrium Iteratively Nested Keyway (BLINK) and Multi-Locus Mixed Model (MLMM) implemented in the GAPIT package in R. Before association testing, population structure was assessed by principal component analysis (PCA), with the first three principal components included as covariates alongside a kinship matrix. MLMM iteratively incorporates significant markers as covariates, simultaneously detecting multiple loci contributing to phenotypic variation (Segura et al., 2012). BLINK filters redundant markers using linkage disequilibrium (LD) and applies the Bayesian Information Criterion (BIC) for model selection, enhancing statistical power while controlling false positives (Huang et al., 2019). For multiple testing correction, a Bonferroni adjustment at α = 0.05 was applied based on 289,569 SNPs, yielding a genome-wide significance threshold of *p* < 1.726 × 10^-7^ (−log_10_*p* = 6.76). GWAS results were visualized using Manhattan and quantile–quantile (QQ) plots generated with the “qqman” R package (Turner, 2018). Significant SNPs were annotated by assigning open reading frames (ORFs) within a ±150-kb window around each SNP as candidate genes.

### 2.6 Statistical analysis

Variation in PHS among rice genetic resources was evaluated using one-way analysis of variance (ANOVA) in R version 4.3.1 software. When ANOVA results were significant (p < 0.05), group comparisons were performed using Duncan’s multiple range test through the “agricolae” R package (de Mendiburu, 2023) to identify statistically significant differences among genetic resources.

### 2.7 Allelic distribution analysis of *SDR4* and *qPH7* loci

Two-locus haplotype analysis of *SDR4* and *qPH7* was performed using PCR amplification with Solg™ e-Taq DNA Polymerase (SolGent, Daejeon, Korea) (**Table 2**). For the SDR4-SacII marker (Sugimoto et al., 2010), thermal cycling conditions included an initial denaturation at 94 °C for 5 min; 35 cycles of 94 °C for 20 s, 55 °C for 25 s, and 72 °C for 1 min; with a final extension at 72 °C for 5 min. For the PH_1_13 marker (Lee et al., 2023), the same conditions were used except for the annealing step at 54 °C for 45 s. PCR products for *SDR4* and *qPH7* were digested with the restriction enzymes *Sac*II and *Dde*I, respectively. Digested products were separated on a 1.5% agarose gel and stained with SYBR™ Safe DNA Gel Stain (Thermo Fisher Scientific, Waltham, MA, USA). Bands were visualized using the DAVINCH Gel Imager CG-550 (DAVINCH-K, Seoul, Korea).

**Table 2:** Marker information used for the identification of PHS-related genes, *SDR4* and *qPH7*.

QTL/gene	Chr	Marker	Sequence (5’-3’)	Restriction enzyme	Reference
*SDR4*	7	SDR4-SacII	F: GTGTCGGTGGTGGTCGTC R: CGAGAACCCCTTGCATGTCT	*SacII*	(Sugimoto et al., 2010)
*qPH7*	7	PH_1_13	F: ATCTGTATGACTTAAGGCACG R: ACTAAACTGTGCTAAATTGCG	*DdeI*	(Lee et al., 2023)

## 3 Results

### 3.1 Pre-harvest sprouting phenotypic variation in diverse rice genetic resources

**Figure 1:**
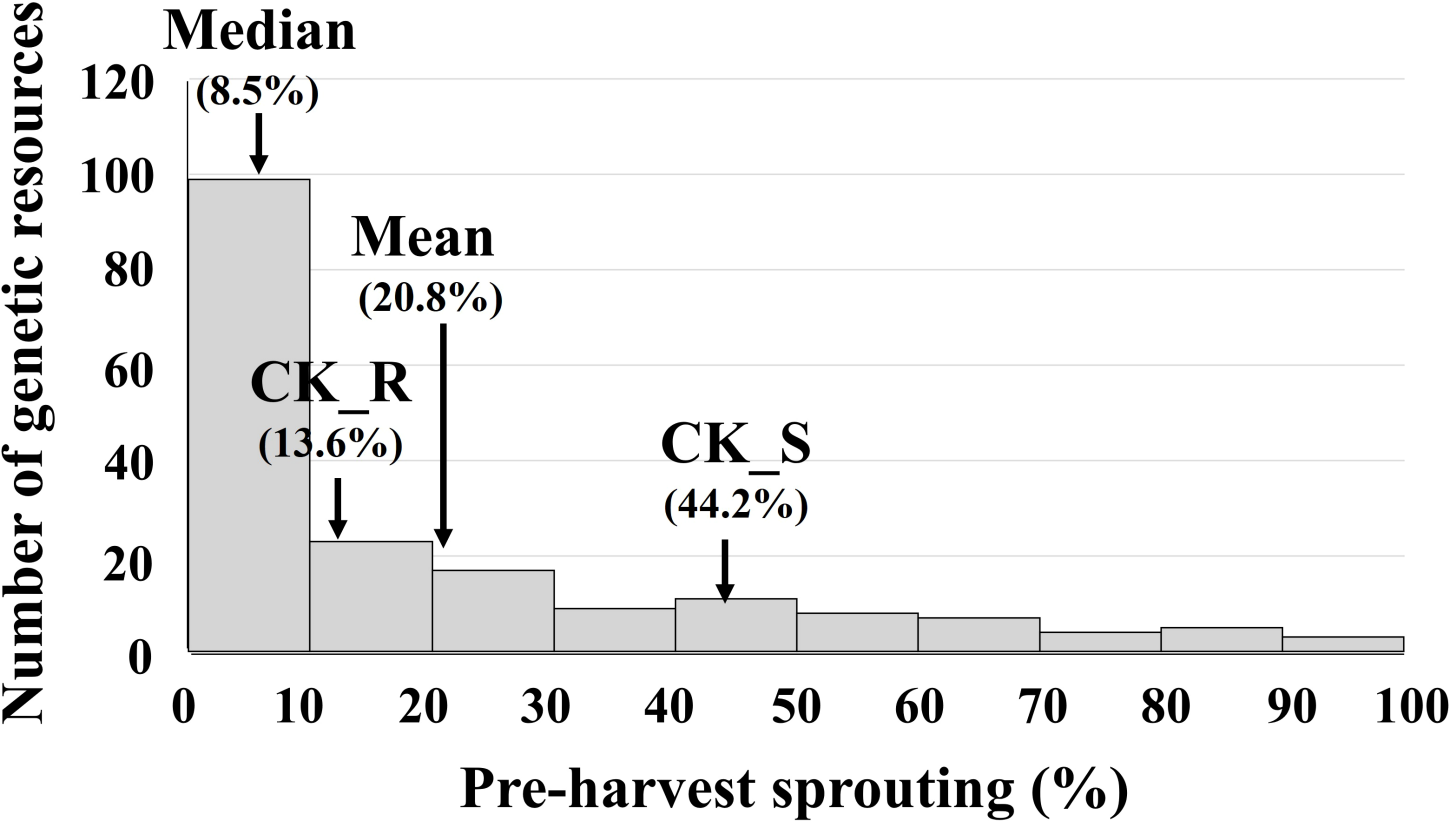
Distribution of PHS rates in 182 rice genetic resources, with the mean, median and PHS rate of the reference resistance control cultivar Joun (CK_R) and the susceptible control cultivar Jopyeong (CK_S) indicated. PHS, pre-harvest sprouting.

PHS was evaluated in 182 rice genetic resources using the predefined criteria (**Table 1**). PHS rates ranged from 0% to 95.7%, with an average of 20.8 ± 25.2% (**Figure 1**). The distribution was right-skewed (skewness = 1.36; kurtosis = 0.87). The median value was 8.5%, indicating that most rice genetic resources exhibited relatively low sprouting levels. The resistant control cultivar, Joun, showed an average PHS rate of 13.61 ± 4.23%, whereas the susceptible control, Jopyeong, exhibited a significantly higher rate of 44.23 ± 5.30%.

**Figure 2:**
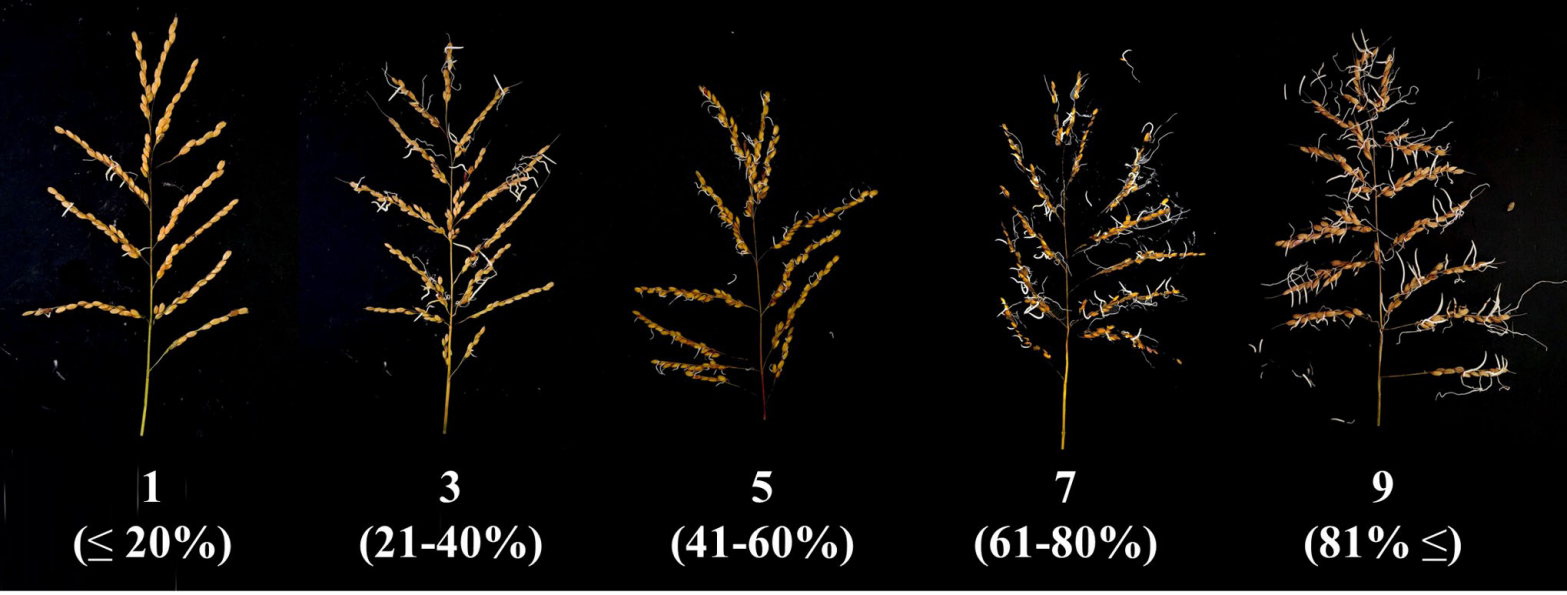
Representative images illustrating pre-harvest sprouting (PHS) severity across selected classification degrees (1–9) in 182 rice genetic resources. The displayed genetic resources are *japonica* rice cultivars: degree 1, Koshihikari; degree 3, Jonong; degree 5, Chinnong; degree 7, Goami; and degree 9, Istiqbol.

PHS rates were classified into five degrees according to the established evaluation criteria (**Table 1**; **Figure 2**). Among the 182 genetic resources, 20.9% were classified as susceptible (degree 5, 7, or 9), while the remaining 79.1% were classified as resistant (degree 1 or 3). The resistant and susceptible control cultivars corresponded to degree 1 and degree 5, respectively. To evaluate PHS variation among ecotypes within the population, the genetic resources were grouped into five ecotype categories and evaluated for PHS levels based on predefined criteria (**Supplementary Figure S1**). *Japonica* (group I) and *Indica* (group II) showed the widest PHS variation, with Indica showing a lower median value than that of *Japonica*. In contrast, *Admixed* (group III) predominantly exhibited low PHS rates.

### 3.2 Genotypic profiling and population structure of rice genetic resources

Overall, 289,569 SNPs were analyzed across 182 resources using an SNP chip and next-generation sequencing (NGS) (**Supplementary Table S1**). SNP distribution varied across the 12 rice chromosomes, with an average of 24,131 SNPs per chromosome. Chromosome 1 showed the highest number (37,852), whereas chromosome 12 had the lowest (16,551). SNP density also differed among chromosomes, averaging 1.32 SNPs/Mb (**Supplementary Figure S2**). Chromosome 12 exhibited the highest density (1.66 SNPs/Mb), while chromosomes 2 and 3 showed the lowest (1.09 SNPs/Mb).

A phylogenetic analysis of 182 rice genetic resources was performed using genome-wide SNP data to assess genetic relationships and population structure (**Supplementary Figures S3A, B**). The neighbor-joining tree analysis revealed five distinct ecotype groups based on genetic similarity: (i) *Japonica*, (ii) *Indica*, (iii) *Admixed*, (iv) *Aus*, and (v) *Aromatic* (**Supplementary Figure S3A**). These groups reflect unique genetic backgrounds and ecological adaptations, with pronounced divergence between *Japonica* and *Indica*.

Genetic structure was further validated through PCA (**Supplementary Figure S3B**), which supported the same five-group clustering pattern. PC1 and PC2 explained 60.38% and 4.63% of the total genetic variation, respectively. The PCA results showed clear genetic differentiation among ecotypes, providing complementary evidence to the phylogenetic analysis. This grouping establishes the basis for interpreting phenotypic variation in traits such as PHS resistance.

### 3.3 Genome-wide association analysis for pre-harvest sprouting resistance

**Figure 3:**
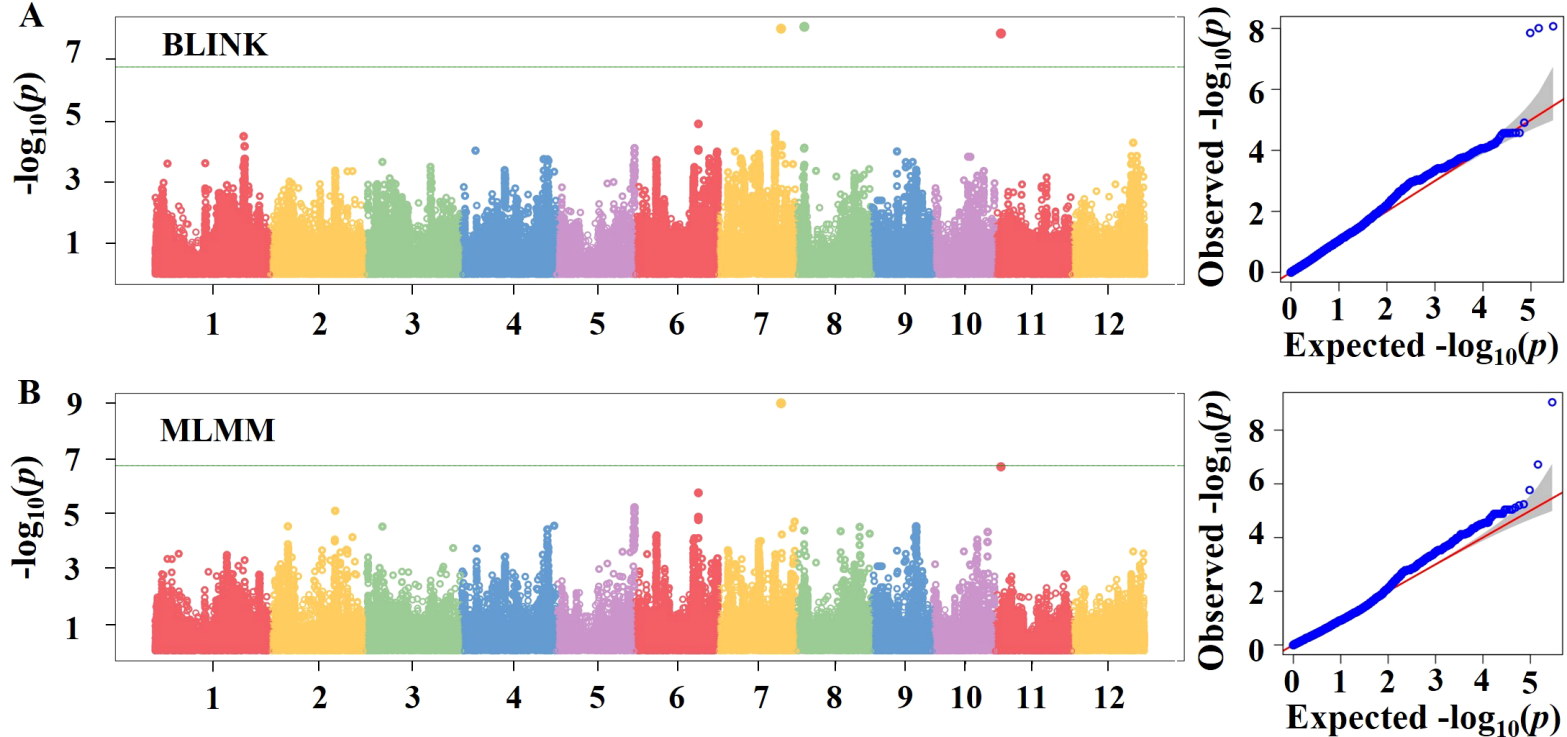
GWAS of PHS in 182 rice genetic resources. **(A)** Quantile–quantile (QQ) plot and Manhattan plot generated using the BLINK model. In the Manhattan plot, the x-axis represents the genomic positions across the 12 rice chromosomes, and the y-axis represents −log_10_(*P*) values. The green horizontal line indicates the genome-wide significance threshold (−log_10_(*P*) = 6.76). **(B)** QQ plot and Manhattan plot generated using the MLMM model, with axes and the genome-wide significance threshold defined as in **(A)**. GWAS, genome-wide association study; PHS, pre-harvest sprouting; BLINK, Bayesian information and linkage disequilibrium iteratively nested keyway; MLMM, multi-locus mixed model; QQ, quantile–quantile.

GWAS were performed using genotypic and PHS rate data from 182 rice genetic resources to identify SNPs associated with PHS resistance. The GAPIT package in R was used to implement the BLINK and MLMM models. As illustrated in the Manhattan and quantile–quantile (QQ) plots (**Figure 3**), SNP associations were evaluated across the 12 rice chromosomes using a genome-wide significance threshold of −log_10_(*P*) = 6.76, based on a Bonferroni correction using 289,569 SNPs. The QQ plots showed that the observed *p*-values closely followed the expected distribution, indicating minimal genomic inflation and adequate control of population structure. BLINK identified significant SNPs on chromosomes 7, 8, and 11 (**Figure 3A**), while MLMM detected lead SNPs on chromosomes 7 and 11 (**Figure 3B**). The SNPs on chromosomes 7 and 11 were detected at identical loci in both models, whereas the SNP on chromosome 8 was unique to BLINK. These lead SNPs on chromosomes 7, 8, and 11 were considered QTLs and designated as *qRPH7, qRPH8, and qRPH11*, respectively (**Table 3**).

**Table 3:** Summary of QTLs associated with PHS identified through GWAS.

QTLs	SNP	Chr	Pos (bp)	-log_10_(*p*)	MAF	PVE	Model
*qRPH7*	AX-115841304	7	23,799,472	8.01	0.08	43.69	BLINK
*qRPH8*	AX-275910905	8	2,882,518	8.07	0.35	32.84	“
*qRPH11*	AX-115796079	11	2,374,434	7.85	0.13	13.07	“
*qRPH7*	AX-115841304	7	23,799,472	9.04	0.08	80.00	MLMM
*qRPH11*	AX-115796079	11	2,374,434	6.72	0.13	33.89	“

Chr, chromosome; MAF, minor allele frequency; PVE, phenotype variance explained; QTL, quantitative trait loci; GWAS, genome-wide association study; PHS, pre-harvest sprouting; BLINK, Bayesian information and linkage disequilibrium iteratively nested keyway; MLMM, multi-locus mixed model; SNP, single nucleotide polymorphism.

*qRPH7* showed strong statistical significance in both BLINK and MLMM, with –log_10_(*p*) values of 8.01 and 9.04 and phenotypic variance explained (PVE) values of 43.69% and 80.00%, respectively. *qRPH11* showed –log_10_(*p*) values of 7.52 in the BLINK model and 6.72 in the MLMM model, the latter being slightly below but close to the predefined significance threshold (6.76). Despite this marginal significance, *qRPH11* consistently explained a substantial proportion of phenotypic variance, with PVE values ranging from 13.07% to 33.89% across the two models. Based on its consistent detection and large effect size, *qRPH11* was retained as a candidate QTL and selected for subsequent analyses. *qRPH8* was detected exclusively by BLINK, with a –log_10_(*p*) value of 8.07 and a PVE of 32.84%. Despite differences between the models, both BLINK and MLMM consistently identified three QTLs significantly associated with PHS resistance.

**Figure 4:**
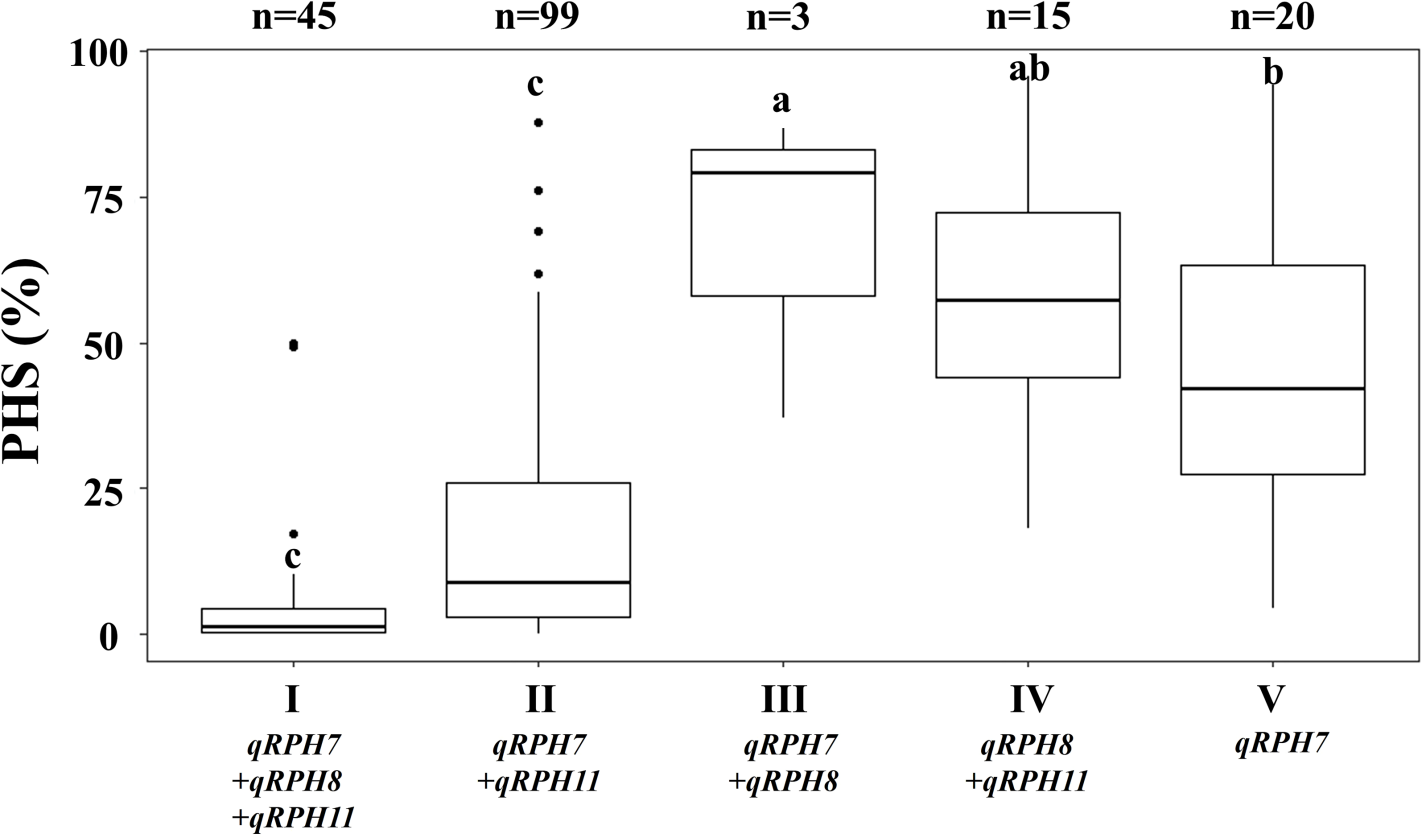
Pre-harvest sprouting (PHS) rate of rice genetic resources grouped based on allelic combinations at three QTLs (*qRPH7, qRPH8, qRPH11*). Different letters denote significant differences among groups based on Duncan’s multiple range test.

To assess the effects of the identified QTLs, the 182 rice genetic resources were classified into five groups (I-V) based on their QTL combinations, and PHS was confirmed (**Figure 4**). Group I included all three QTLs—*qRPH7*, *qRPH8*, and *qRPH11*. Groups II–IV each contained two QTLs: Group II possessed *qRPH7* and *qRPH11*; Group III had *qRPH7* and *qRPH8*; and Group IV included *qRPH8* and *qRPH11*. Group V included genetic resources carrying only *qRPH7*. Group I showed the lowest mean PHS rate, and Groups I and II exhibited significantly lower PHS rates than those of other groups.

### 3.4 Candidate genes and genotypic effect of identified quantitative trait loci

**Figure 5:**
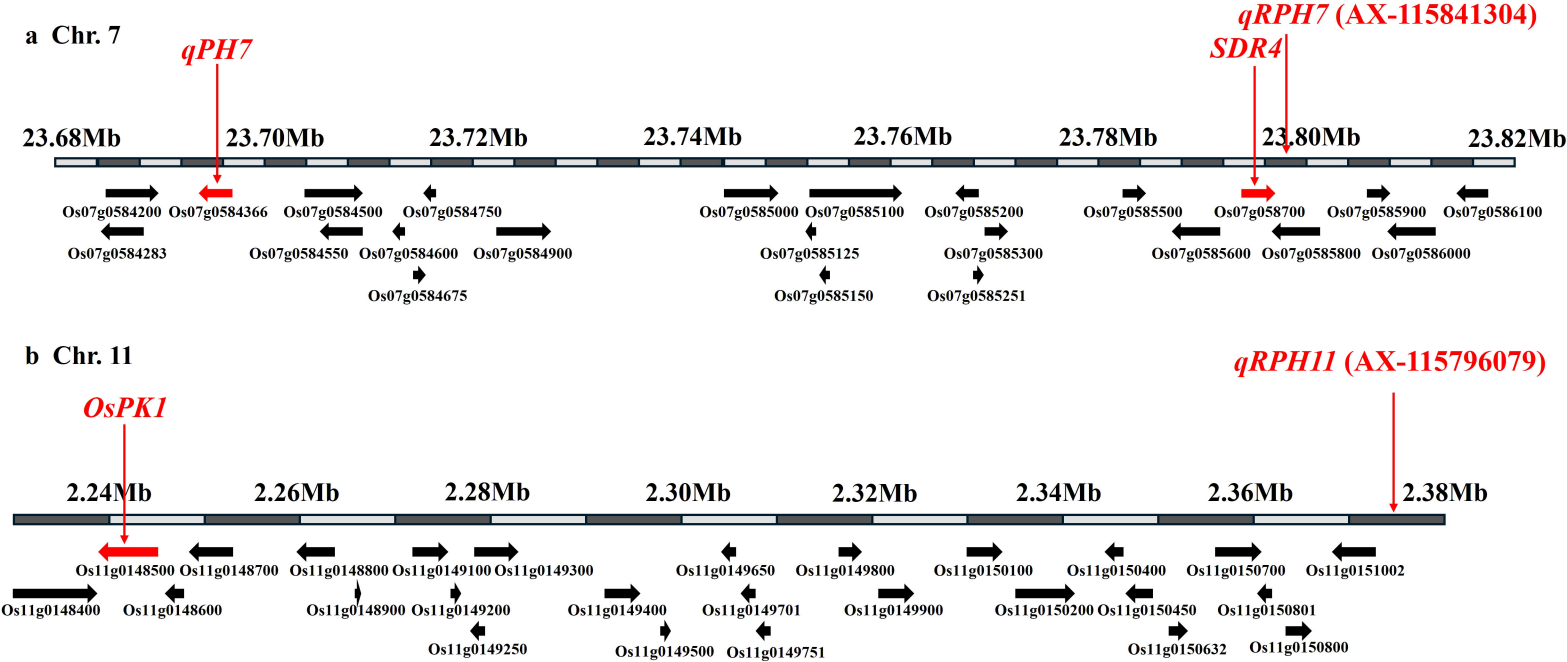
**(A)***qRPH7* on chromosome 7 (red arrow) and surrounding genes, with previously reported PHS-related genes, *SDR4* and *qPH7*, highlighted in red. **(B)***qRPH11* on chromosome 11 (red arrow) and surrounding genes, with previously reported ABA/GA-related gene, *OsPK1*, highlighted in red. PHS, pre-harvest sprouting; ABA, Abscisic acid; GA, gibberellins.

To identify candidate genes related to PHS resistance, ORFs located within ±150 kb of the three QTLs (*qRPH7, qRPH8*, and *qRPH11*) identified through GWAS were examined. The analysis prioritized genes annotated in the Rice Annotation Project database and functionally related to PHS, germination, seed dormancy, and ABA/GA signaling pathways (**Supplementary Table S2**). Within the *qRPH7* interval, two major loci linked to PHS resistance—*SDR4* and *qPH7*—were identified (**Figure 5A**). Additionally, the *qRPH11* region harbored *OsPK1*, a gene implicated in hormonal regulation related to PHS resistance (**Figure 5B**). In contrast, no annotated ORFs with known roles in PHS, seed dormancy, or ABA/GA signaling were identified within the ±150 kb region flanking qRPH8. Thus, leaving the functional candidate gene(s) underlying this QTL unresolved.

Among the 182 rice genetic resources, 167 carrying *qRPH7* were selected to analyze the genotypes of the candidate loci *SDR4* and *qPH7* (**Supplementary Figure S4**). Consequently, 53 genetic resources carried *SDR4*, 42 carried *qPH7*, and 39 possessed both genes. In contrast, 14 carried *SDR4* alone, three carried only *qPH7*, and 111 carried neither locus.

Among the 167 rice genetic resources excluding group IV (**Figure 4**), those carrying *qRPH7* were classified into four combination types (A–D) based on the presence or absence of *SDR4* and *qPH7*, and their PHS rates were compared (**Table 4**). Type D, comprising 111 rice genetic resources lacking both *SDR4* and *qPH7*, was selected for additional analysis to evaluate the effects of other QTLs. Type A (*SDR4 + qPH7*) exhibited the lowest mean PHS rate (2.0 ± 2.5%), followed by Type C (*qPH7*, 3.8 ± 5.5%), Type B (*SDR4*, 6.1 ± 5.6%), and Type D (neither locus), which had the highest rate at 24.8 ± 24.7%. The overall mean PHS rate was 17.5 ± 22.7%. According to PHS classification criteria, Types A, B, C, and the overall mean were categorized as degree 1 (≤ 20%), whereas Type D corresponded to degree 3 (21–40%).

**Table 4:** Pre-harvest sprouting (PHS) rates in 167 rice genetic resources with *qRPH7*, grouped by *SDR4* and *qPH7* combinations.

Genotypic combination type of *qRPH7*	No. of plants	PHS (%)	Degree
*qRPH7*			
A. *SDR4* + *qPH7*	39	2.0 ± 2.5	1
B. *SDR4*	14	6.1 ± 5.6	1
C. *qPH7*	3	3.8 ± 5.5	1
D. *None*	111	24.8 ± 24.7	3
Total	167	17.5 ± 22.7	1

To further dissect the genetic basis of PHS resistance, the 111 rice genetic resources classified as Type D in **Table 4** (lacking both SDR4 and qPH7) were analyzed based on the presence or absence of qRPH8 and qRPH11 (**Table 5**). Based on the presence or absence of *qRPH8* and *qRPH11*, these resources were further classified into four types (a–d), and their PHS rates were compared. Among these groups, type a (*qRPH11* + *qRPH8*) showed a mean PHS rate of 20.6 ± 26.4%, type b (*qRPH11*) exhibited 18.3 ± 19.5%, type c (*qRPH8*) recorded a markedly higher rate of 67.8 ± 27.1%, and type d (*none*) showed 46.0 ± 26.9%. The overall average PHS rate was 24.8 ± 24.7%, corresponding to degree 3 (21–40%). Based on the PHS classification criteria, type b was categorized as degree 1, type a as degree 3, type d as degree 5, and type c as degree 7. Duncan’s multiple range test (p < 0.05) revealed significant differences in PHS rates among types: types c and d were grouped as “a”, while types a and b were grouped as “b”, indicating that groups carrying *qRPH11* exhibited significantly lower PHS rates.

**Table 5:** Pre-harvest sprouting (PHS) rates in 111 Type D rice genetic resources (lacking *SDR4* and *qPH7*), classified by combinations of *qRPH11* and *qRPH8*.

Subtype of D carrying *qRPH7*	No. of plants	PHS (%)	Degree
a. *qRPH11 + qRPH8*	5	20.6 ± 26.4^b^	3
b. *qRPH11*	83	18.3 ± 19.5^b^	1
c. *qRPH8*	3	67.8 ± 27.1^a^	7
d. *None*	20	46.0 ± 26.9^a^	5
Total	111	24.8 ± 24.7	3

In total, 111 rice genetic resources classified as Type D in **Table 4** (lacking both *SDR4* and *qPH7*) were further subdivided into four subtypes based on the presence or absence of *qRPH11* and *qRPH8*, and their PHS rates were compared.

### 3.5 Integrative modeling of quantitative trait loci-based resistance mechanisms in rice

**Figure 6:**
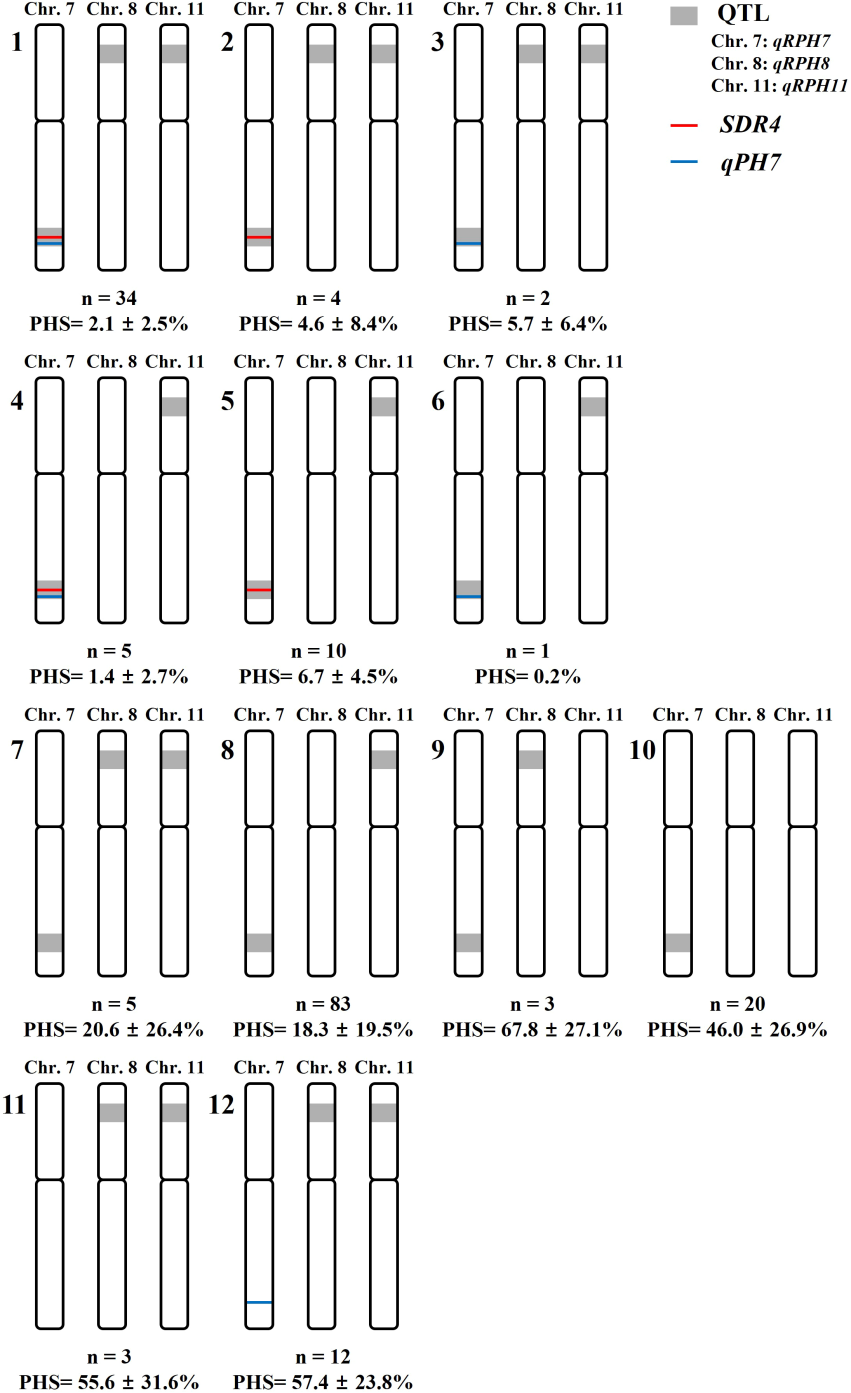
Classification of 182 rice genetic resources into 12 allelic combinations based on two previously reported loci and three QTLs.

The effects of different QTL combinations on PHS resistance were evaluated (**Figure 6**). Strong resistance was observed in genetic resources carrying *qRPH7, qRPH8*, and *qRPH11* simultaneously (groups 1–3), which showed low PHS rates of 2.1 ± 2.5%, 4.6 ± 8.4%, and 5.7 ± 6.4%, respectively. Similarly, genetic resources harboring *qRPH7* and *qRPH11* (groups 4–6) demonstrated very strong resistance, with PHS rates of 1.4 ± 2.7%, 6.7 ± 4.5%, and 0.2%, respectively. In contrast, group 7, which was comparable to groups 1–3 but lacked either *SDR4* or *qPH7*, and group 8, which was comparable to groups 4–6 but lacked *SDR4* or *qPH7*, exhibited moderate resistance, with PHS rates of 20.6 ± 26.4% and 18.3 ± 19.5%, respectively. However, both groups exhibited considerable phenotypic variation. By comparison, group 9 (*qRPH7* and *qRPH8*) and group 10 (*qRPH7* alone) exhibited high PHS rates of 67.8 ± 27.1% and 46.0 ± 26.9%, respectively. Similarly, group 11 (*qRPH8* and *qRPH11*) showed a high PHS rate of 55.6 ± 31.6%. In group 12, the additional of *qRPH7* along with *qRPH8* and *qRPH11* did not reduce the PHS rate, which remained high at 57.4 ± 23.8%.

## 4 Discussion

### 4.1 Quantitative trait loci identification through genome-wide association study

Rice (*Oryza sativa*) is a critical global crop, but its productivity and quality are highly susceptible to environmental threats such as PHS. While previous studies identify loci associated with PHS resistance, most have focused on specific cultivars, leaving the broader molecular mechanisms unresolved.

In the GWAS analysis using the BLINK model, three putative QTLs—*qRPH7, qRPH8*, and *qRPH11*—were detected (**Figure 3**, **Table 3**). Among these, *qRPH7* and *qRPH11* were also identified by MLMM, with *qRPH7* accounting for a high proportion of phenotypic variance 33.89~80.00% (**Table 3**). This overlap between models highlights the robustness of these loci. Conversely, *qRPH8* was detected only by BLINK, and its inconsistent phenotypic association suggests a limited contribution to PHS resistance.

### 4.2 Quantitative trait loci effects on pre-harvest sprouting within tested plants

In rice genetic resources, three major QTLs—*qRPH7*, *qRPH8*, and *qRPH11*—that exhibit additive cumulative effects on PHS resistance (**Figure 4**). The lowest mean PHS incidence (4.5 ± 10.4%) was observed in Group I, which harbors all three QTLs, suggesting that the combination of these loci is highly effective in enhancing resistance. In contrast, Group II (*qRPH7* and *qRPH11*) demonstrated a lower PHS incidence (16.1 ± 18.6%) and was classified as resistant; however, the wide phenotypic variance observed in this group suggests potential influence from genetic background or environmental factors. The effect of a single QTL was observed only in Group V, which contains *qRPH7* alone. Since no genetic resource lines individually carried *qRPH8* or *qRPH11*, their single effects could not be evaluated. While *qRPH7* exhibited the highest PVE, its solitary presence in Group V did not confer significant resistance.

Although *qRPH7* and *qRPH11* were consistently detected across GWAS models, the wide phenotypic variation among *qRPH7* carriers indicates strong inter-locus interactions. *qRPH11* acts additively to enhance *qRPH7*-mediated resistance, whereas *qRPH8* functions as a context-dependent antagonist that modulates resistance expression.

### 4.3 Identification of candidate genes within quantitative trait loci regions

To identify candidate genes influencing PHS, ORFs within the three significant QTLs were functionally annotated based on a comprehensive review. The analysis focused on ORFs annotated in the Rice Annotation Project database (https://rapdb.dna.affrc.go.jp/) that are associated with PHS, general germination, seed dormancy, and ABA/GA signaling pathways (**Supplementary Table S2**). Within the genomic region surrounding *qRPH7* (± 150 kb), the presence of two major loci associated with PHS resistance—*SDR4* and *qPH7*—were confirmed (**Figure 5**). Among the 167 rice genetic resources carrying *qRPH7*, 56 (33.5%) possessed at least one of these two major loci, *SDR4* or *qPH7* (**Supplementary Figure S4**). Within this group, 14 carried only *SDR4*, 3 carried only *qPH7*, and 39 possessed both loci.

Within the fine-mapped *qPH7* region, *Os07g0584366* exhibited nearly ninefold higher expression in the PHS-resistant donor Wandoaengmi6 compared to susceptible japonica cultivars, supporting its association with *qPH7*-linked resistance (Lee et al., 2023). In addition, the seed dormancy regulator *Sdr4* has been shown to contribute substantially to natural variation in dormancy through both haplotype variation and differences in expression level. The Kasalath allele (*Sdr4-k*) confers deeper dormancy and stronger resistance to PHS, whereas the Nipponbare allele (*Sdr4-n*) is associated with reduced dormancy and increased susceptibility (Sugimoto et al., 2010). Previous studies have further reported that higher expression of *Sdr4* is associated with suppressed germination and enhanced seed dormancy, indicating that *Sdr4* function depends not only on allelic variation but also on transcriptional regulation (Chen et al., 2021). Moreover, *Sdr4* acts downstream of the ABA-responsive transcription factor *OsVP1*, functioning as part of a regulatory network that integrates ABA signaling during seed maturation. Together, these results show that candidate genes located within the *qRPH7* region are functionally associated with seed dormancy and ABA-mediated signaling pathways.

*qRPH7* is located within a genomic region where the previously reported PHS-resistance QTL and genes, *qPH7* and *SDR4*, are positioned in proximity within the ±150 kb interval. However, in the present study, PHS resistance was observed in rice genetic resources carrying *qRPH7* despite the absence of both *SDR4* and *qPH7*. This finding suggests that *qRPH7* may contribute to PHS resistance independently of these previously characterized genes and further raises the strong possibility that additional PHS-associated candidate genes are present within this region. Therefore, the PHS resistance associated with *qRPH7* cannot be sufficiently explained solely by the effects of *SDR4* or *qPH7*. Future studies will aim to identify novel PHS-related candidate genes within the *qRPH7* interval through gene expression profiling and transcriptome analyses. In addition, the genetic effects and potential interaction mechanisms among *SDR4*, *qPH7*, and *qRPH7* in regulating PHS resistance will be further investigated.

Within the ±150 kb genomic region surrounding *qRPH11*, 52 ORFs were identified, among which *OsPK1*—a gene known to regulate the balance between ABA and GA—was the only locus associated with PHS or seed germination (**Figure 5B**). In rice, *OsPK1* is a metabolism-related gene that contributes to growth regulation and environmental adaptation (Zhang et al., 2012). By modulating the ABA/GA balance, *OsPK1* integrates hormonal signaling and functions as a molecular link between stress responses and growth suppression. Consistent with this role, knockout mutants of *OsPK1* (*ospk1*) were reported to accumulate higher levels of abscisic acid (ABA) while suppressing gibberellin (GA) biosynthesis, resulting in an altered ABA/GA balance, enhanced dormancy, and increased oxidative stress. In contrast, no annotated ORFs with known functions associated with PHS, seed dormancy, or ABA/GA signaling were identified within the ±150 kb region flanking *qRPH8*.

### 4.4 Synergistic and antagonistic effects of quantitative trait loci combinations on pre-harvest sprouting resistance in rice

Among the 167 genetic resources carrying *qRPH7*, four QTL combination types were classified based on the presence of *SDR4, qPH7*, or both, with some combinations significantly associated with reduced PHS rates (**Table 4**). Genetic resources in types A, B, and C—each carrying either *SDR4*, *qPH7*, or both—consistently exhibited low PHS rates, indicating strong resistance. In contrast, type D, which possesses *qRPH7* but lacks *SDR4* and *qPH7*, demonstrated a markedly broader distribution and higher mean PHS rates. This divergence suggests that *qRPH7* alone is insufficient to confer stable resistance and highlights the significant individual and combined contributions of *SDR4* and *qPH7* in enhancing seed dormancy and suppressing PHS.

In the Type D subtype, the roles of *qRPH8* and *qRPH11* were investigated to further dissect the genetic architecture underlying PHS resistance in the absence of *SDR4* and *qPH7* (**Table 5**). Among the four genotypic combinations evaluated (types a–d), types a and b, carrying *qRPH11*, consistently exhibited lower PHS rates compared to those that lack this locus (types c and d). Type b, carrying *qRPH11* alone, exhibited the lowest PHS rate (18.3 ± 19.5%) and was classified as degree 1 resistance, suggesting that *qRPH11* positively contributes to resistance, either independently or in combination. Type c, which carries only *qRPH8*, exhibited the highest PHS rate (67.8 ± 27.1%), suggesting that in certain genetic backgrounds, *qRPH8* may function as an epistatic gene, suppressing the effects of other resistance loci or even promoting susceptibility. *qRPH8* exhibits epistatic interactions that antagonize, rather than enhance, PHS resistance, complicating its functional interpretation given its variable phenotypic expression. Overall, *qRPH8* is inferred to act as a negative regulator of other PHS resistance–associated QTLs, thereby increasing the PHS rate.

Duncan’s multiple range test results further support the significant contribution of *qRPH11* to PHS resistance, with types a and b forming distinct statistical groups compared to those of types c and d. These findings suggest an additive effect of *qRPH11*, while the apparent lack of beneficial effect from *qRPH8* raises concerns about its utility in breeding programs and warrants further functional characterization.

The *indica* ecotype BALA displayed a PHS rate of 18.0% despite the absence of the *qRPH7* locus, suggesting that alternative genetic factors contribute to PHS resistance independently of *qRPH7*. Developing a segregating population from this genetic resource would facilitate further investigation of the underlying mechanisms. **Figure 4** illustrates two genetic resources in Group I that appear as outliers, exhibiting high PHS rates of 49.2% and 49.8%, respectively. These genetic resources lacked *SDR4* and *qPH7*, despite carrying *qRPH7* and *qRPH11*, suggesting that the absence of *SDR4* and *qPH7* may have a greater effect on PHS susceptibility than the presence of *qRPH7* and *qRPH11*. Therefore, functional analysis incorporating *SDR4* and *qPH7* will be essential to elucidate the genetic interactions among these loci.

Overall, these results highlight how *qRPH7*, *qRPH8*, and *qRPH11* interact synergistically and antagonistically in modulating PHS resistance. To further contextualize these findings within the broader genetic framework, all 12 possible QTL–loci combinations were analyzed (**Figure 6**), which revealed the complex genetic architecture underlying PHS resistance. Based on these observations, multi-locus combinations were then examined to determine how additive and epistatic interactions collectively shape PHS resistance.

### 4.5 Complex genetic architecture of pre-harvest sprouting resistance

PHS resistance was evaluated using 12 allelic combinations derived from three QTLs (*qRPH7, qRPH8*, and *qRPH11*) and two loci (*SDR4, qPH7*) (**Figure 6**). The results indicate that both the additive effects of individual QTLs and their genetic interactions are essential for determining PHS resistance. Strong resistance was observed in groups harboring all three QTLs (Groups 1–3) and in those carrying *qRPH7* together with *qRPH11* (Groups 4–6). In contrast, Groups 7 and 8, which had the same QTL combinations as Groups 1–3 and 4–6, respectively, but lacked *SDR4* and *qPH7*, exhibited lower average resistance and greater variation, indicating that the effect of *qRPH7* depends on the presence of *SDR4* and *qPH7*. A comparison between Groups 8 and 10 further supports this finding: Group 10 (*qRPH7* alone) exhibited high susceptibility, while Group 8 (*qRPH7* + *qRPH11* without *SDR4* and *qPH7*) showed overall resistance, suggesting that *qRPH11* acts additively to enhance the effect of *qRPH7*.

More specifically, Group 7 comprised three highly resistant genetic resources (≤ 20% PHS) and two susceptible (>40%), while Group 8 included 83 genetic resources, of which 55 were highly resistant (≤ 20%), 17 moderately resistant (≤ 40%), and 11 susceptible. These findings suggest that in the absence of *SDR4* and *qPH7*, *qRPH11*, in combination with *qRPH7*, contributes to resistance. However, some genetic resources remain susceptible, indicating that minor QTLs or background genetic variation may also influence PHS. The most notable finding was the antagonistic epistasis of *qRPH8*. Among combinations lacking *SDR4* and *qPH7*, groups carrying *qRPH7* with *qRPH8* or *qRPH8* with *qRPH11* (Groups 9, 11, 12) exhibited high PHS rates. In contrast, resistance was observed when all three QTLs (*qRPH7, qRPH8, qRPH11*) were present (Groups 1–3, 7). These findings suggest that *qRPH8* suppresses the effect of *qRPH7* or *qRPH11* when present individually, leading to susceptibility, but when all three QTLs are combined, this antagonistic effect is neutralized. Thus, *qRPH8* may function as an antagonistic regulator, modulating the effects of other major QTLs rather than acting only as a minor contributor.

Collectively, these findings indicate that PHS resistance is regulated by complex interactions among *qRPH7–SDR4–qPH7, qRPH11*, and *qRPH8*, rather than by a single major locus. Specifically, *qRPH7* functions in an *SDR4*- and *qPH7*-dependent manner and is further enhanced by *qRPH11*, while *qRPH8* exerts antagonistic epistasis by suppressing or modifying the effects of the other loci. These findings highlight that PHS is a typical polygenic trait, governed by additive effects and complex interactions among multiple loci.

Our results show a complex genetic interplay among multiple loci contributing to PHS resistance in rice. The consistent effects of *SDR4*, *qPH7*, and *qRPH11* suggest that combining these loci through marker-assisted selection could substantially enhance resistance. In contrast, the effects of *qRPH8* are inconsistent or adverse, highlighting the need for careful interpretation of its role. Future studies should investigate potential epistatic interactions among these loci and account for environmental influences that may affect the phenotypic expression of resistance.

In breeding, these findings provide a practical framework for improving PHS resistance in rice. We propose a targeted pyramiding strategy incorporating *SDR4*, *qPH7*, and *qRPH11* to develop rice cultivars with enhanced PHS resistance. The inclusion of *qRPH8* in breeding programs should be carefully considered, as its phenotypic effects are inconsistent. Using these validated loci in marker-assisted selection may accelerate the development of resilient varieties, particularly under humid and warm conditions that increase PHS risk. Moreover, integrating genotype-by-environment interaction analyses will be essential to ensure stable resistance across diverse cultivation settings.

## Data Availability

The raw data supporting the conclusions of this article will be made available by the authors, without undue reservation.

## Glossary

PHS: Pre-harvest sprouting
GWAS: Genome-wide association study
QTLs: Quantitative trait loci
ABA: Abscisic acid
GA: Gibberellins
ROS: Reactive oxygen species
MAF: Minor allele frequency
BLINK: Bayesian-information and linkage-disequilibrium iteratively nested keyway
MLMM: Multi-Locus mixed model
LD: Linkage disequilibrium
BIC: Bayesian information criterion
QQ: Quantile–quantile
ORFs: Open reading frames
ANOVA: One-way analysis of variance
NGS: Next-generation sequencing
PCA: Principal component analysis
SNP: Single-nucleotide polymorphism
