# Supplementary material for: A new genetic architecture for PHS resistance in rice: deciphering the epistatic interactions of three major QTL
Source: Front Plant Sci. 2026 Feb 11;17:1778741. doi: 10.3389/fpls.2026.1778741 (PMC12932557; doi:10.3389/fpls.2026.1778741)
Supplement: Supplementary file 1 [file DataSheet1.docx]

**Supplementary Tables**

**Supplementary Table S1.** Summary of SNPs used for GWAS analysis in the rice genetic resource

| Chr | Length  (bp, IRGSP-1.0) | SNP position  (bp, start-end) | No. of SNPs | SNPs/Mb |
| --- | --- | --- | --- | --- |
| 1 | 43,270,923 | 1,695−43,269,728 | 37,852 | 860.3 |
| 2 | 35,937,250 | 1,933−35,935,363 | 33,110 | 919.7 |
| 3 | 36,413,819 | 2,137−36,402,891 | 33,400 | 902.7 |
| 4 | 35,502,694 | 5,098−3,549,193 | 22,160 | 615.6 |
| 5 | 29,958,434 | 28,278−29,766,349 | 21,579 | 719.3 |
| 6 | 31,248,787 | 40,333−31,236,749 | 24,680 | 771.3 |
| 7 | 29,697,621 | 4,996−29,682,463 | 21,120 | 704.0 |
| 8 | 28,443,022 | 10,132−28,439,244 | 20,976 | 723.3 |
| 9 | 23,012,720 | 38,929−22,938,953 | 18,284 | 795.0 |
| 10 | 23,207,287 | 4,426−23,205,938 | 18,890 | 787.1 |
| 11 | 29,021,106 | 25,385−29,011,352 | 20,967 | 698.9 |
| 12 | 27,531,856 | 51,004−27,529,256 | 16,551 | 591.1 |
| Total (aver.) | 373,245,519 | - | 289,569  (24,131) | (757.4) |

**Supplementary Table S2.** List of ORFs within ±150 kb of the peak SNPs

| Chr | MSU ID | RAP ID | Function |
| --- | --- | --- | --- |
| 7 | *LOC_Os07g39470* | *Os07g0583600* | gibberellin response modulator protein, putative, expressed |
|  | *LOC_Os07g39480* | *Os07g0583700* | WRKY87, expressed |
|  | *LOC_Os07g39490* | *Os07g0583800* | expressed protein |
|  | *LOC_Os07g39500* | *Os07g0583900* | expressed protein |
|  | *LOC_Os07g39510* | *Os07g0584000* | yippee zinc-binding protein, putative, expressed |
|  | *LOC_Os07g39520* | *Os07g0584100* | STE_PAK_Ste20_Slob_Wnk.5 - STE kinases include homologs to sterile 7, sterile 11 and sterile 20 from yeast, expressed |
|  | *LOC_Os07g39530* | *Os07g0584200* | BTBN16 - Bric-a-Brac, Tramtrack, Broad Complex BTB domain with non-phototropic hypocotyl 3 NPH3 domain, expressed |
|  | *LOC_Os07g39550* | *Os07g0584366* | expressed protein |
|  | *LOC_Os07g39560* | *Os07g0584500* | RNA recognition motif-containing protein, putative, expressed |
|  | *LOC_Os07g39570* | *Os07g0584750* | expressed protein |
|  | *LOC_Os07g39590* | *Os07g0584900* | Spotted leaf 11, putative, expressed |
|  | *LOC_Os07g39600* | *-* | transposon protein, putative, CACTA, En/Spm sub-class |
|  | *LOC_Os07g39610* | *-* | transposon protein, putative, CACTA, En/Spm sub-class, expressed |
|  | *LOC_Os07g39620* | *Os07g0585000* | C2 domain-containing protein, putative, expressed |
|  | *LOC_Os07g39630* | *Os07g0585100* | calcium-binding protein, putative, expressed |
|  | *LOC_Os07g39640* | *Os07g0585200* | LTPL64 - Protease inhibitor/seed storage/LTP family protein precursor, expressed |
|  | *LOC_Os07g39650* | *Os07g0585251* | hypothetical protein |
|  | *LOC_Os07g39660* | *-* | hypothetical protein |
|  | *LOC_Os07g39670* | *-* | retrotransposon protein, putative, Ty1-copia subclass, expressed |
|  | *LOC_Os07g39680* | *Os07g0585500* | UV-induced protein uvi15, putative, expressed |
|  | *LOC_Os07g39690* | *Os07g0585600* | GCN5-related N-acetyltransferase, putative, expressed |
|  | *LOC_Os07g39700* | *Os07g0585700* | expressed protein |
|  | *LOC_Os07g39710* | *Os07g0585800* | NADH dehydrogenase iron-sulfur protein 4, mitochondrial precursor, putative, expressed |
|  | *LOC_Os07g39720* | *Os07g0585900* | expressed protein |

**Supplementary Table S2.** List of ORFs within ±150 kb of the peak SNPs (continued)

| Chr | MSU ID | RAP ID | Function |
| --- | --- | --- | --- |
| 7 | *LOC_Os07g39730* | *Os07g0586000* | ZCW7, putative, expressed |
|  | *LOC_Os07g39740* | *Os07g0586100* | GDSL-like lipase/acylhydrolase, putative, expressed |
|  | *LOC_Os07g39750* | *Os07g0586200* | GDSL-like lipase/acylhydrolase, putative, expressed |
|  | *LOC_Os07g39760* | *Os07g0586332* | expressed protein |
|  | *LOC_Os07g39770* | *Os07g0586400* | expressed protein |
|  | *LOC_Os07g39780* | *Os07g0586500* | SUMO-activating enzyme subunit 2, putative, expressed |
| 8 | *LOC_Os08g05250* | *-* | retrotransposon protein, putative, Ty3-gypsy subclass, expressed |
|  | *LOC_Os08g05260* | *-* | retrotransposon protein, putative, unclassified |
|  | *LOC_Os08g05270* | *Os08g0148100* | retrotransposon protein, putative, unclassified, expressed |
|  | *LOC_Os08g05280* | *Os08g0148200* | oxidoreductase family, NAD-binding Rossmann fold containing protein, expressed |
|  | *LOC_Os08g05290* | *Os08g0148300* | receptor-like protein kinase 5 precursor, putative, expressed |
|  | *LOC_Os08g05300* | *Os08g0148400* | expressed protein |
|  | *LOC_Os08g05310* | *Os08g0148566* | expressed protein |
|  | *LOC_Os08g05320* | *Os08g0148600* | expressed protein |
|  | *LOC_Os08g05330* | *-* | expressed protein |
|  | *LOC_Os08g05340* | *Os08g0149000* | expressed protein |
|  | *LOC_Os08g05360* | *-* | transposon protein, putative, unclassified, expressed |
|  | *LOC_Os08g05370* | *Os08g0149333* | conserved hypothetical protein |
|  | *LOC_Os08g05380* | *-* | expressed protein |
|  | *LOC_Os08g05390* | *-* | transposon protein, putative, CACTA, En/Spm sub-class, expressed |
|  | *LOC_Os08g05400* | *-* | transposon protein, putative, CACTA, En/Spm sub-class, expressed |
|  | *LOC_Os08g05410* | *-* | expressed protein |

**Supplementary Table S2.** List of ORFs within ±150 kb of the peak SNPs (continued)

| Chr | MSU ID | RAP ID | Function |
| --- | --- | --- | --- |
| 8 | *LOC_Os08g05420* | *Os08g0149900* | transposon protein, putative, CACTA, En/Spm sub-class, expressed |
|  | *LOC_Os08g05430* | *-* | transposon protein, putative, CACTA, En/Spm sub-class, expressed |
|  | *LOC_Os08g05440* | *Os08g0150150* | NB-ARC domain-containing protein, expressed |
|  | *LOC_Os08g05450* | *-* | transposon protein, putative, unclassified, expressed |
|  | *LOC_Os08g05460* | *Os08g0150500* | OsFBL40 - F-box domain and LRR-containing protein, expressed |
|  | *LOC_Os08g05470* | *Os08g0150600* | expressed protein |
|  | *LOC_Os08g05480* | *Os08g0150700* | OsFBX261 - F-box domain-containing protein, expressed |
|  | *LOC_Os08g05490* | *Os08g0150800* | tRNA synthetase, putative, expressed |
|  | *LOC_Os08g05500* | *-* | retrotransposon protein, putative, unclassified |
|  | *LOC_Os08g05510* | *Os08g0151000* | MYB family transcription factor, putative, expressed |
|  | *LOC_Os08g05520* | *Os08g0151100* | myb-like DNA-binding domain containing protein, putative, expressed |
|  | *LOC_Os08g05530* | *Os08g0151400* | LSM domain-containing protein, expressed |
|  | *LOC_Os08g05540* | *Os08g0151500* | expressed protein |
|  | *LOC_Os08g05550* | *Os08g0151600* | expressed protein |
|  | *LOC_Os08g05560* | *Os08g0151700* | zinc finger, C3HC4 type domain containing protein, expressed |
|  | *LOC_Os08g05570* | *Os08g0151800* | monodehydroascorbate reductase, putative, expressed |
|  | *LOC_Os08g05580* | *Os08g0151900* | aquaporin protein, putative, expressed |
|  | *LOC_Os08g05590* | *Os08g0152000* | aquaporin protein, putative, expressed |
|  | *LOC_Os08g05600* | *Os08g0152100* | aquaporin protein, putative, expressed |
| 11 | *LOC_Os11g05080* | *Os11g0148200* | powdery mildew resistant protein 5, putative, expressed |
|  | *LOC_Os11g05090* | *Os11g0148300* | peptidyl-prolyl isomerase, putative, expressed |
|  | *LOC_Os11g05100* | *Os11g0148400* | nucleolar GTPase, putative, expressed |

**Supplementary Table S2.** List of ORFs within ±150 kb of the peak SNPs (continued)

| Chr | MSU ID | RAP ID | Function |
| --- | --- | --- | --- |
| 11 | *LOC_Os11g05110* | *Os11g0148500* | pyruvate kinase, putative, expressed |
|  | *LOC_Os11g05120* | *Os11g0148600* | expressed protein |
|  | *LOC_Os11g05130* | *Os11g0148700* | PHD-finger family protein, expressed |
|  | *LOC_Os11g05140* | *Os11g0148800* | expressed protein |
|  | *LOC_Os11g05150* | *Os11g0148900* | hydroxyproline-rich glycoprotein family protein, putative, expressed |
|  | *LOC_Os11g05160* | *Os11g0149100* | DNA-binding protein, putative, expressed |
|  | *LOC_Os11g05170* | *Os11g0149200* | expressed protein |
|  | *LOC_Os11g05180* | *Os11g0149300* | expressed protein |
|  | *LOC_Os11g05190* | *Os11g0149400* | phytosulfokines precursor, putative, expressed |
|  | *LOC_Os11g05200* | *Os11g0149500* | zinc finger, C3HC4 type domain containing protein, expressed |
|  | *LOC_Os11g05210* | *-* | retrotransposon protein, putative, unclassified, expressed |
|  | *LOC_Os11g05220* | *-* | transposon protein, putative, unclassified, expressed |
|  | *LOC_Os11g05230* | *Os11g0149800* | zinc finger, C3HC4 type domain containing protein, expressed |
|  | *LOC_Os11g05240* | *Os11g0149900* | PAN domain-containing protein At5g03700 precursor, putative, expressed |
|  | *LOC_Os11g05250* | *-* | hypothetical protein |
|  | *LOC_Os11g05260* | *Os11g0150100* | phosphoglycerate mutase, putative, expressed |
|  | *LOC_Os11g05270* | *-* | hypothetical protein |
|  | *LOC_Os11g05280* | *-* | expressed protein |
|  | *LOC_Os11g05290* | *Os11g0150400* | Stress-responsive A/B Barrel domain-containing protein, expressed |
|  | *LOC_Os11g05300* | *Os11g0150450* | RING-H2 finger protein, putative, expressed |
|  | *LOC_Os11g05310* | *Os11g0150632* | expressed protein |
|  | *LOC_Os11g05320* | *Os11g0150700* | AGC_PVPK_like_kin82y.1 - ACG kinases include homologs to PKA, PKG and PKC, expressed |

**Supplementary Table S2.** List of ORFs within ±150 kb of the peak SNPs (continued)

| Chr | MSU ID | RAP ID | Function |
| --- | --- | --- | --- |
| 11 | *LOC_Os11g05340* | *-* | transposon protein, putative, unclassified, expressed |
|  | *LOC_Os11g05350* | *-* | expressed protein |
|  | *LOC_Os11g05360* | *-* | RCLEA9 - Root cap and Late embryogenesis related family protein precursor, putative, expressed |
|  | *LOC_Os11g05370* | *Os11g0151300* | 60S ribosomal protein L26-1, putative, expressed |
|  | *LOC_Os11g05380* | *Os11g0151400* | cytochrome P450, putative, expressed |
|  | *LOC_Os11g05390* | *Os11g0151500* | transporter, major facilitator family, putative, expressed |
|  | *LOC_Os11g05394* | *Os11g0151600* | nucampholin, putative, expressed |
|  | *LOC_Os11g05400* | *Os11g0151700* | Ser/Thr protein phosphatase family protein, putative, expressed |
|  | *LOC_Os11g05410* | *Os11g0151800* | Ser/Thr protein phosphatase family protein, putative, expressed |
|  | *LOC_Os11g05420* | *Os11g0151900* | expressed protein |
|  | *LOC_Os11g05430* | *-* | expressed protein |
|  | *LOC_Os11g05440* | *-* | expressed protein |
|  | *LOC_Os11g05450* | *Os11g0152025* | expressed protein |
|  | *LOC_Os11g05460* | *Os11g0152150* | expressed protein |
|  | *LOC_Os11g05470* | *Os11g0152500* | RCN1 Centroradialis-like1 homogous to TFL1 gene; contains Pfam profile PF01161: Phosphatidylethanolamine-binding protein, expressed |
|  | *LOC_Os11g05480* | *Os11g0152700* | transcription factor, putative, expressed |
|  | *LOC_Os11g05490* | *Os11g0153000* | expressed protein |
|  | *LOC_Os11g05494* | *Os11g0153100* | expressed protein |
|  | *LOC_Os11g05500* | *-* | conserved hypothetical protein |
|  | *LOC_Os11g05510* | *-* | carbonic anhydrase family protein, putative, expressed |
|  | *LOC_Os11g05520* | *-* | bifunctional monodehydroascorbate reductase and carbonic anhydrasenectarin-3 precursor, putative, expressed |

**Supplementary Table S2.** List of ORFs within ±150 kb of the peak SNPs (continued)

| Chr | MSU ID | RAP ID | Function |
| --- | --- | --- | --- |
| 11 | *LOC_Os11g05530* | *Os11g0153300* | expressed protein |
|  | *LOC_Os11g05540* | *Os11g0153400* | rhoGAP domain-containing protein, expressed |
|  | *LOC_Os11g05550* | *Os11g0153500* | expressed protein |
|  | *LOC_Os11g05552* | *Os11g0153600* | signal recognition particle 54 kDa protein, chloroplast precursor, putative, expressed |
|  | *LOC_Os11g05556* | *Os11g0153700* | signal recognition particle 54 kDa protein, putative, expressed |
|  | *LOC_Os11g05562* | *Os11g0153800* | 40S ribosomal protein S25, putative, expressed |

**Supplementary Figures**


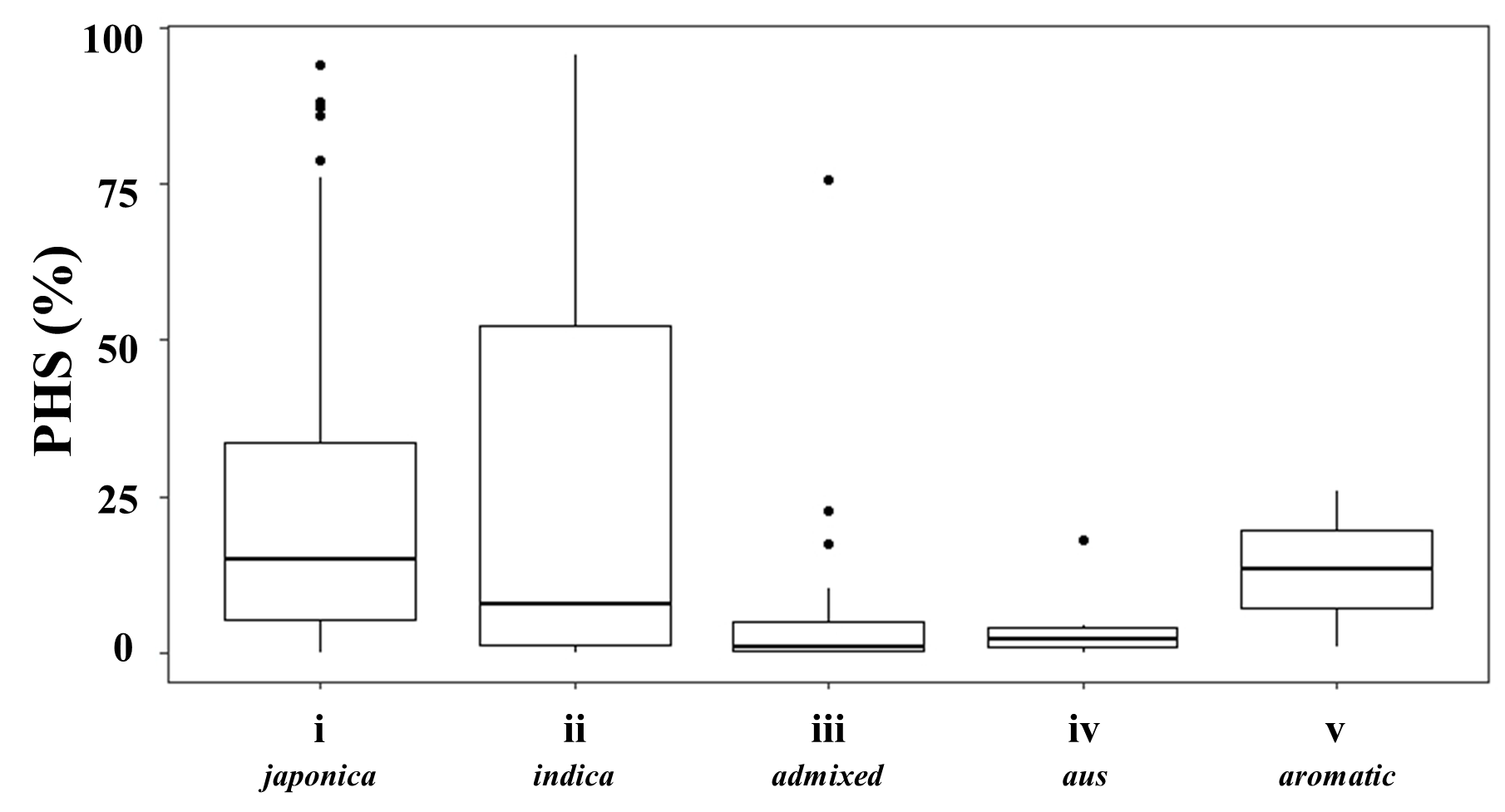


**Supplementary Figure S1.** Pre-harvest sprouting (PHS) rates of 182 rice genetic resources grouped by ecotype: i, *Japonica*; ii, *Indica*; iii, *Admixed*; iv, *Aus*; v, *Aromatic*. The figure illustrates variations in germination rates among ecotypes and highlights differences in PHS resistance across groups


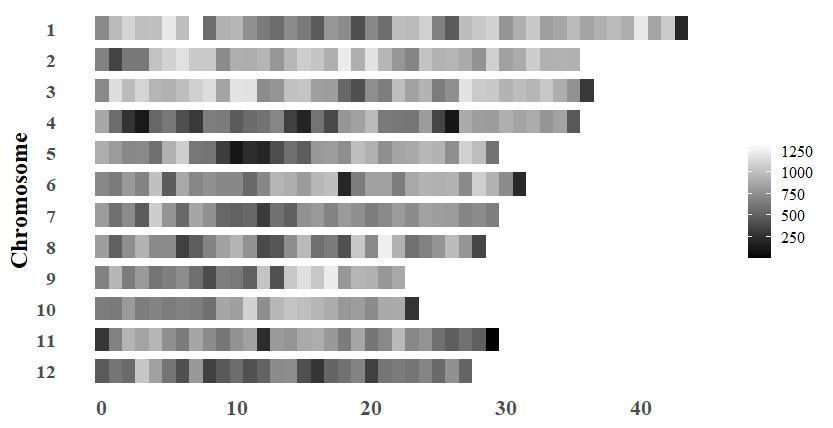


**Supplementary Figure S2.** SNP density on each chromosome, represented as the number of SNPs per Mb. SNP, single-nucleotide polymorphism


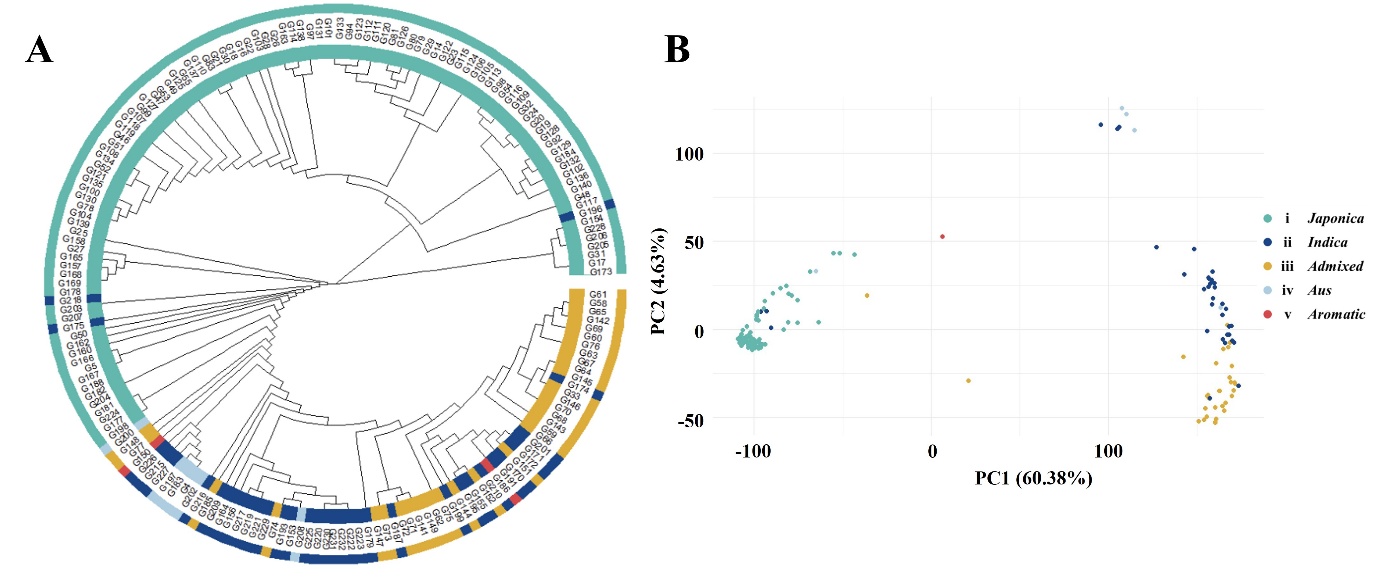
**Supplementary Figure S3.** Genetic diversity among 182 rice genetic resources illustrated using two complementary approaches. a Phylogenetic tree constructed using the neighbor-joining method based on genome-wide SNPs. b PCA showing genetic clustering based on SNP variation. Ecotype groups are color-coded as follows: i, *Japonica*; ii, *Indica*; iii, *Admixed*; iv, *Aus*; v, *Aromatic*. SNP, single-nucleotide polymorphism; PCA, principal component analysis


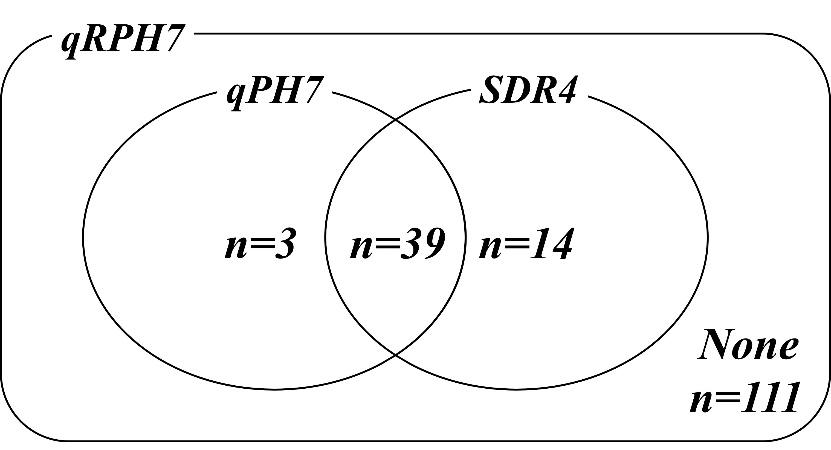


**Supplementary Figure S4.** Venn diagram showing the genotypic overlap between *qPH7* and *SDR4* among 167 rice genetic resources carrying *qRPH7*
